# Supplementary material for: Systematic review of social determinants of childhood immunisation in low- and middle-income countries and equity impact analysis of childhood vaccination coverage in Nigeria
Source: PLoS One. 2024 Mar 6;19(3):e0297326. doi: 10.1371/journal.pone.0297326 (PMC10917251; doi:10.1371/journal.pone.0297326)
Supplement: S1 Table — (DOCX) [file pone.0297326.s002.docx]

**S1 Table. Routine vaccination schedule of under 1-year-old children in Nigeria.**

| Vaccine | Schedule |
| --- | --- |
| BCG (Bacille Calmette-Guérin) | Birth |
| Hepatitis B (birth dose) | Birth |
| Oral polio | Birth  6, 10, 14 weeks |
| DTP-HepB-Hib (diphtheria, tetanus, pertussis, hepatitis B and Haemophilus influenzae type B) | 6, 10, 14 weeks |
| Pneumococcal conjugate | 6, 10, 14 weeks |
| Inactivated polio | 14 weeks |
| Measles first dose | 9 months |
| Meningitis A conjugate | 9 months |
| Yellow fever | 9 months |
