# Supplementary material for: Systematic review of social determinants of childhood immunisation in low- and middle-income countries and equity impact analysis of childhood vaccination coverage in Nigeria
Source: PLoS One. 2024 Mar 6;19(3):e0297326. doi: 10.1371/journal.pone.0297326 (PMC10917251; doi:10.1371/journal.pone.0297326)
Supplement: S2 Table — (DOCX) [file pone.0297326.s003.docx]

**S4 Table Vaccination coverage and vaccination card usage rates in Nigeria.** Vaccination card usage rates and vaccination coverage among children aged 12-23 months in Nigeria, at the national level and disaggregated by urban and rural areas of residence.

|  | National coverage  (% and 95% CI)  n = 6059 | Urban coverage  (% and 95% CI)  n1 = 2100 | Rural coverage  (% and 95% CI)  n2 = 3959 |
| --- | --- | --- | --- |
| Vaccination card | 48.6  (46.3, 50.8) | 62.9  (59.1, 66.6) | 39.2  (36.7, 41.7) |
|  | | | |
| Vaccine | | | |
| BCG | 66.7  (64.4, 68.8) | 83.3  (80.3, 85.9) | 55.8  (53.0, 58.7) |
| Polio 1^st^ dose | 72.8  (70.7, 74.8) | 80.3  (77.3, 83.0) | 67.9  (65.2, 70.6) |
| Polio 2^nd^ dose | 66.8  (64.7, 68.8) | 76.1  (72.9, 79.0) | 60.7  (58.0, 63.3) |
| Polio 3^rd^ dose | 48.0  (46.0, 50.1) | 56.4  (53.2, 59.6) | 42.5  (39.9, 45.2) |
| Polio – all three doses | 47.2  (45.2, 49.3) | 55.8  (52.6, 58.9) | 41.7  (39.0, 44.3) |
| No doses of polio | 26.4  (24.4, 28.4) | 18.9  (16.3, 21.8) | 31.7  (28.7, 34.0) |
| DTP 1^st^ dose | 64.8  (62.6, 66.9) | 80.8  (77.8, 83.5) | 54.3  (51.7, 57.0) |
| DTP 2^nd^ dose | 57.8  (55.6, 59.9) | 74.3  (71.1, 77.3) | 47.0  (44.4, 49.6) |
| DTP 3^rd^ dose | 50.7  (48.4, 53.0) | 68.3  (64.7, 71.7) | 39.2  (36.5, 41.9) |
| DTP – all three doses | 50.1  (47.8, 52.4) | 67.9  (64.3, 71.3) | 38.4  (35.8, 41.1) |
| No doses of DTP | 34.7  (32.6, 36.8) | 18.8  (16.2, 21.8) | 45.0  (42.4, 47.7) |
| Measles one dose | 53.9  (51.7, 56.0) | 68.8  (65.5, 71.9) | 44.1  (41.6, 46.6) |
| Basic vaccinations (all) | 31.2  (29.4, 33.1) | 44.2  (41.0, 47.5) | 22.7  (20.7, 24.8) |
| No basic vaccination doses | 19.5  (17.7, 21.4) | 11.2  (9.2, 13.7) | 24.9  (22.4, 27.6) |
