# Supplementary material for: Systematic review of social determinants of childhood immunisation in low- and middle-income countries and equity impact analysis of childhood vaccination coverage in Nigeria
Source: PLoS One. 2024 Mar 6;19(3):e0297326. doi: 10.1371/journal.pone.0297326 (PMC10917251; doi:10.1371/journal.pone.0297326)
Supplement: S3 Table — (DOCX) [file pone.0297326.s004.docx]

**S5. Interaction effects.**

S5A Table: Interaction terms between place of residence and household wealth
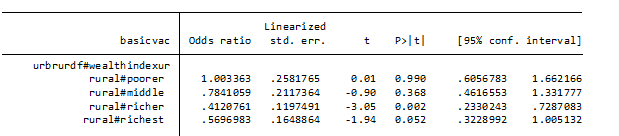


S5B Table: AORs of basic vaccination coverage in Nigeria by household wealth in rural areas in comparison to urban areas

| Characteristics | Adjusted odds ratio | 95% confidence interval | p value |
| --- | --- | --- | --- |
| Rural – poorest | 0.88 | 0.56 – 1.37 | 0.560 |
| Rural – poorer | 0.88 | 0.60 – 1.28 | 0.499 |
| Rural – middle | 0.69 | 0.47 – 1.00 | 0.049 |
| Rural – richer | 0.36 | 0.24 – 0.53 | <0.001 |
| Rural – richest | 0.50 | 0.35 – 0.72 | <0.001 |
