# Supplementary material for: Systematic review of social determinants of childhood immunisation in low- and middle-income countries and equity impact analysis of childhood vaccination coverage in Nigeria
Source: PLoS One. 2024 Mar 6;19(3):e0297326. doi: 10.1371/journal.pone.0297326 (PMC10917251; doi:10.1371/journal.pone.0297326)
Supplement: S4 Table — Vaccine coverage estimates for Nigeria in 2018 based on DHS, WUENIC (WHO-UNICEF), administrative, and official country sources. (DOCX) [file pone.0297326.s005.docx]

**S6 Table: Vaccine coverage.** Vaccine coverage estimates for Nigeria in 2018 based on DHS, WUENIC (WHO-UNICEF), administrative, and official country sources.

| Source | Vaccine coverage (%) | | | |
| --- | --- | --- | --- | --- |
|  | BCG | Measles | DTP 3 | Polio 3 |
| DHS | 67 | 54 | 50 | 47 |
| WUENIC | 67 | 54 | 56 | 56 |
| Administrative – provider estimates | 90 | 87 | 95 | 95 |
| Official country estimates | 75 | 63 | 58 | 58 |
