# Supplementary material for: Systematic review of social determinants of childhood immunisation in low- and middle-income countries and equity impact analysis of childhood vaccination coverage in Nigeria
Source: PLoS One. 2024 Mar 6;19(3):e0297326. doi: 10.1371/journal.pone.0297326 (PMC10917251; doi:10.1371/journal.pone.0297326)
Supplement: S1 Fig — The Preferred Reporting Items for Systematic Reviews and Meta Analyses (PRISMA) flow diagram of articles’ identification, screening, eligibility, and inclusion in the systematic review is illustrated. (DOCX) [file pone.0297326.s006.docx]

**S2 Figure. PRISMA flowchart.** The Preferred Reporting Items for Systematic Reviews and Meta Analyses (PRISMA) flow diagram of articles’ identification, screening, eligibility, and inclusion in the systematic review is illustrated.

**Screening**

**Included**

**Eligibility**

**Identification**

Records identified through database searching MEDLINE

(n = 155)

Records excluded

(n = 2)

Reason: duplication

Records screened by title and abstract

(n = 158)

Records excluded

(n = 103)

Reasons: Irrelevant or fell into exclusion categories

Full-text articles assessed for eligibility

(n = 55)

Studies included in qualitative synthesis

(n = 49)

Full-text articles excluded

(n = 6)

Reasons:

not relevant (n = 3)

not focused on LMICs (n = 2)

data analysed too old to be relevant (n = 1)

Additional records identified through other sources

(n = 5)
